# Taking stock of existing evidence and closing evidence gaps – Reflections from the National Institute for Health and Care Excellence (NICE)

**DOI:** 10.25646/6502

**Published:** 2020-06-04

**Authors:** Kay Nolan

**Affiliations:** National Institute for Health and Care Excellence, Centre for Guidelines

The National Institute for Health and Care Excellence (NICE) has been applying the principles of evidence-based medicine to public health problems for over twelve years. This application is in effect the practice of evidence-based public health (EBPH) [1]. NICE as an organisation applies common principles for the development of all its evidence-based guidelines regardless of topic, one of which is the use of the ‘best available evidence’. In the development of any public health guideline, NICE takes stock of the existing evidence base.

Infrequently is it practical to assess the effectiveness of interventions for complex public health problems by traditional gold standard randomised control trials. The importance of context, wider determinants, subpopulation variability and the role of the non-human biology element often mean that specific high quality evidence is unavailable. There is often a lack of good outcome studies answering the specific ‘what works’ question. There are often fewer studies still that answer the questions what works for whom and under what circumstances? The evidence is also often too imprecise to determine the relationship between the intervention and the outcome.

The public health evidence base is rarely perfect, if it were it would likely negate the need for, or change the approach and role of organisations such as NICE. Perhaps more importantly the question asked in the evidence interrogation at NICE is ‘What is the best available evidence to answer the specific question being addressed?’ We know public health questions may benefit from exploration of a broader range of evidence including that which is further down the hierarchy of evidence. By taking a transparent and systematic approach and pulling from evidence from a range of disciplines, NICE has been able to review and synthesise evidence for complex public health problems [2].

The evidence used to generate NICE guidelines is diverse and consists not only of the traditional scientific published evidence but also stakeholder comments, grey literature, real world evidence and expert testimony [3]. The definition of evidence included in the application of EBPH at NICE has, and continues to, evolve over time, what remains consistent is some assessment of the quality of that evidence particularly its biases.

This presentation will aim to describe the approach taken by NICE in interrogating the evidence base to develop national public health guidelines. The key challenges and some solutions will be illustrated through examples from evidence synthesis to support guidelines for non-communicable diseases.
